# Long-Term Clinical and Immunological Effects of Repeated Mesenchymal Stem Cell Injections in Patients With Progressive Forms of Multiple Sclerosis

**DOI:** 10.3389/fneur.2021.639315

**Published:** 2021-05-31

**Authors:** Panayiota Petrou, Ibrahim Kassis, Ariel Ginzberg, Michel Halimi, Nour Yaghmour, Oded Abramsky, Dimitrios Karussis

**Affiliations:** Multiple Sclerosis Center/Neuroimmunology Unit, Department of Neurology, The Agnes-Ginges Center for Neurogenetics, Hadassah University Hospital, Jerusalem, Israel

**Keywords:** multiple sclerosis, stem cell, mesenchymal stem cell, progressive MS, clinical trial

## Abstract

**Background:** Mesenchymal stem cells (MSC) were shown to possess immunomodulatory and neurotrophic effects. Our previous trials, have shown that intrathecal (IT) and intravenous (IV) administration of MSCs were safe and provided indications of beneficial clinical effects.

**Methods:** This is an open prospective study to evaluate the safety and the long-term clinical and immunological effects of multiple injections of autologous MSCs in 24 patients with active-progressive MS. At inclusion, the mean age of the patients was 47.0 ± 9.22, and the mean EDSS score was 6.75 ± 0.68 (range: 5.5–7.5). Patients were initially treated with 1 ×10^6^ MSCS/kg of body weight (IT + IV) and subsequently with up to additional eight courses of MSCs, at intervals of 6–12 months. The duration of the trial was 4 years.

**Results:** No serious, treatment-related adverse events were observed during the follow-up period. Twenty-two of the 24 patients were either stable or improved at the last follow-up visit. Ten patients had a lower than baseline EDSS at the last follow-up (nine were among those who received >2 treatments and one in the subgroup of ≤ 2 treatments, *p* = 0.04). The mean EDSS score reduced from 6.75 ± 0.68 at baseline to 6.42 ± 0.84 at the last visit, during a median follow-up period of 27.8 months (*p* = 0.028). Immunological follow-up showed a transient upregulation of CD4+CD25+FoxP3+ cells and downregulation of the proliferative ability of lymphocytes.

**Conclusions:** Repeated MSC treatments in patients with progressive MS were shown safe at the short/intermediate term and induced clinical benefits (especially in patients treated with >2 injections) that lasted for up to 4 years, paralleled by short-term immunomodulatory effects.

**Clinical Trial Registration:** www.ClinicalTrials.gov, identifier: NCT04823000.

## Introduction

Mesenchymal stem cells (MSCs) are non-hematopoietic stromal cells, which reside mainly in the bone marrow compartment, and also in fat and other tissues. Their classical role is to support hematopoiesis and produce cells of the mesodermal lineage (1). Studies have described additional MSC properties, including immunomodulatory and neurotrophic effects (2–7). In preclinical studies, intravenous (IV) and intrathecal (IT) administration of MSCs has been shown to suppress experimental autoimmune encephalomyelitis (EAE) (3, 7, 8) and support remyelination following spinal trauma, brain ischemia, or induced demyelination (9).

A few small, mostly open-label, clinical trials have reported indications of favorable effects of MSC treatment in stroke, multisystem atrophy, multiple sclerosis (MS), and amyotrophic lateral sclerosis (ALS) (10–18). Whether the observed benefits were mediated by immunomodulatory mechanisms or by neurotrophic and neuroprotective effects remains controversial. Overall, MSCs given intravenously or intrathecally in MS were well-tolerated, with preliminary indications of clinical beneficial effects (12, 13).

In the latter trial (13), based on the data in EAE models (indicating probably two distinct mechanisms of action by the two different routes of MSC administration), a combined intrathecal and intravenous administration was used to maximize the potential therapeutic benefit by accessing the CNS both through the cerebrospinal fluid and the systemic circulation. The injected MSC, labeled with the superparamagnetic iron oxide ferumoxides (Feridex) could be visualized by MRI in the occipital horns of the ventricles, the meninges, subarachnoid space, and spinal cord, indicating a possible migration of the injected MSC to these areas.

In our recently published—first of its kind—phase II double-blind controlled study we examined the efficacy of MSC transplantation in progressive MS (19) and showed that autologous intrathecal MSC transplantation was safe and induced robust clinical beneficial effects. The intrathecal administration was found superior to the intravenous one. In most of the previously reported studies (17, 20, 21), there were signs of fading-off of the beneficial effects by time, with a peak benefit within 1–3 months following the administration of the stem cells.

We report here the results of an open prospective study with multiple intrathecal injections of autologous MSC in 24 patients with progressive forms of MS (secondary progressive, primary progressive, or relapsing progressive), who failed to respond to first and second lines of immunomodulatory treatments.

## Methods/Study Protocol

### Patients

**Figure 1:**
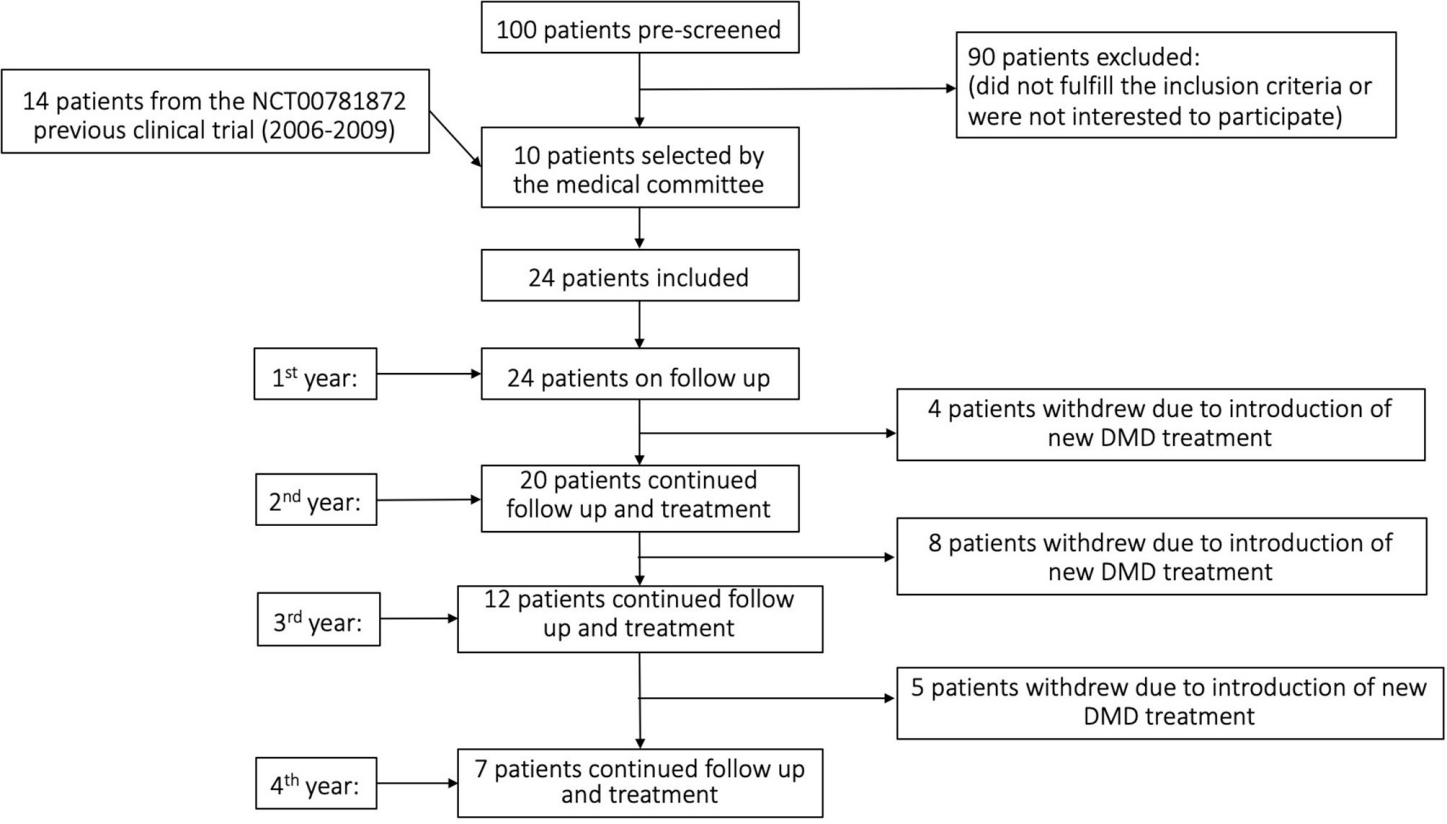
Study flowchart.

Twenty-four patients, 12 males and 12 females [14 from those who participated in our previous clinical trial (13)] were included in this open-label trial, which was originally designed to represent an extension phase of our 2010 study (see study flowchart in Figure 1). In order to formulate a group with at least 24 patients (which would be borderly sufficient to detect significant clinical changes), we received a new license from Hadassah Hospital Ethics committee and the Israel Minsitry of Health to include 10 additional patients. The main aim of the current study was to evaluate the safety (and as secondary aim to detect signals of clinical and immunological effects) of repeated (up to eight) injections of autologous MSC during a period of up to 4 years. The participants suffered from progressive forms of MS (22 with secondary progressive MS and two with primary progressive MS) and were failures to first and second lines of immunomodulatory treatments (as defined in the inclusion criteria). All patients had either deteriorated (by at least 0.5 degree in the EDSS scale for baseline EDSS of >5.0 or 1 degree for lower EDSS scores) during the year preceding their inclusion to our study, or suffered from at least one major relapse accompanied by MRI activity (new lesions, expanding lesions, or gadolinium-enhancing lesions), or two clinical relapses. At inclusion, the mean age of our patients was 47.0 ± 9.22, the mean EDSS score was 6.75 ± 0.68 (range 5.5–7.5), and the mean duration of the disease was 13.4 ± 6.6 years (Table 1: Demographics of the patients). The patients did not receive any immunomodulatory treatment during the period that remained in the trial.

**Table 1:** Demographics of the patients.

**Patients (Gender)**	**Age**	**Years of MS**	**EDSS 1 year before**	**Relapse or MRI activity during last year**	**EDSS at baseline**	**MS type**	**Previous DMDs**
001 (M)	60	21	6.5	R^*^, M^**^	6.5	SPMS	Interferon, glatiramer acetate, mitoxatrone, natalizumab
002 (F)	45	13	5.5		6	SPMS	Interferon, natalizumab
003 (F)	49	17	6	R, M	6	SPMS	Interferon, natalizumab
004 (F)	54	30	6		6.5	SPMS	Glatiramer acetate, fingolimod
005 (F)	47	14	6.5	R, M	6.5	SPMS	Interferon, azathioprine, glatiramer acetate, natalizumab, mycophenolate
006 (F)	48	17	6.5		7	SPMS	Plasmapheresis, rituximab mitoxanthrone, interferon, glatiramer acetate
007 (F)	48	14	7	R, M	7	SPMS	Rituximab, azathioprine, natalizumab, interferon, plasmapheresis
008 (M)	47	11	7		7.5	PPMS	Mitoxathrone
009 (M)	43	10	7.5	R, M	7.5	SPMS	Interferon, natalizumab, mitoxanthrone
010 (M)	47	12	7	R, M	7	SPMS	Mycophenolate, interferon, IVIG
011 (F)	52	8	7	R, M	7	SPMS	HSCT, glatiramer acetate, interferon, natalizumab
012 (M)	27	9	6	R(2), M	6	SPMS	Plasmapheresis, mycophenolate
013 (M)	45	12	7		7.5	SPMS	Interferon, glatiramer acetate, natalizumab
014 (F)	53	7	5.5	R, M	5.5	SPMS	Azathioprine, natalizumab, methyprednisolone monthly pulses
015 (M)	70	6.5	7		7.5	PPMS	Mycophenolate, cyclophosphamide, rituximab
016 (M)	40	7	7	R	7.5	SPMS	Natalizumab, fingolimod
017 (F)	48	7	7		7.5	SPMS	Interferon, natalizumab
018 (M)	48	11	6	R, M	6	SPMS	Mycophenolate, azathioprine, natalizumab
019 (M)	30	9	5.5	R(2), M	5.5	SPMS	Glatiramer acetate, fingolimod, natalizumab
020 (F)	45	19	6		6.5	SPMS	Interferon, natalizumab
021 (M)	56	30	6		6.5	SPMS	Plasmapheresis, interferon, dimethyl fumarate, teriflunomide
022 (M)	30	8	7.0	R, M	7.5	SPMS	Interferon, fingolimod, natalizumab
023 (F)	49	10	6.0	R, M	6.5	SPMS	Interferon, natalizumab
024 (F)	46	19	7		7.5	SPMS	Interferon, azathioprine, mitoxathrone

* *R, relapse of MS during the year prior to inclusion.*

** *M, MRI activity (appearance of new, expanding, or enhancing lesions during the year prior to inclusion)*.

Seven patients received only two treatments, whereas the rest ones were treated with a variable number (3-8) of MSC injections. All 24 patients had a 1 year follow-up, 20 patients remained at follow-up at 2 years, 12 patients at 3 years, and seven patients at 4 years. The patients who stopped the follow-up did so because they expressed their will to start one of the new, disease-modifying drugs for MS that evolve during the time of the trial (Tables 1, **3**). In total, in the whole group of patients, 86 IV and 64 IT MSC injections were performed. Sixty-one out of the total 89 treatments included a combined IT + IV injection of MSC. Less than a third of the injections were not combined ones (IT + IV), either due to unwillingness of the patients to undergo additional lumbar punctures or due to insufficient number of cells.

### Study Design

#### Inclusion Criteria

1. Consenting patients fulfilling the Poser's criteria for definite MS.
2. Age 18–70.
3. Male and female.
4. EDSS 5.5–7.5 (moderate to high disability).
5. Failure to two lines of the currently available registered immunomodulatory treatments [disease-modifying drugs (DMD)] for MS. The lack of response to the treatment was determined by either an increase in EDSS (0.5 degree for EDSS equal or above 5.5 and 1 degree for lower EDSS, confirmed by two evaluations 6 months apart) or the appearance of at least one relapse of MS accompanied by the appearance of new, enlarging or enhancing lesions in the MRI or two relapses, during the year prior to inclusion, under continuous DMD use.

#### Exclusion Criteria

1. Patients who were treated with cytotoxic medications during the last 3 months prior to inclusion (12 months for mitoxanthrone).
2. Patients suffering from significant cardiac, renal, or hepatic failure or any other disease that may risk the patient or interfere with the ability to interpret the results.
3. Patients with active infections.
4. Patients with cognitive decline or inability to understand and sign the informed consent.

#### Treatment Procedures

Bone marrow (BM) was aspirated according to the routine medical center procedure from the patient's iliac crest under local anesthesia and sedation, following testing negativity for HBV, HCV, and HIV. The aspirated BM was transferred immediately to the GMP facility and labeled by the physician or by the attending technical assistant. BM aspirates were transferred from the heparin-containing bone marrow aspiration bags into sterile 50-ml conical tubes (Corning, USA) using two spike tubing sets (Macopharma, USA) and diluted 1:1 (v:v) in Hank's Balanced Salt Solution (HBSS, Sigma-Aldrich), and mono-nuclear cells (MNC) were separated from the total BM inoculum by Ficoll density gradient (1.073 g/ml) centrifugation (GE Healthcare, USA). Diluted BM was transferred to barrier-containing 50-ml tubes (LEUCOSEP™, Greiner-bio one, Germany) prefilled with 15 ml of Ficoll and centrifuged for 10 min, 1,000 × *g*, at 24°C. The MNC layer was removed using sterile pasture pipette (Greiner-bio one, Germany) and transferred to 50-ml sterile tubes and diluted with 30 ml of PBS. Cells were centrifuged twice for 10 min, at 1,000 rpm, 24°C and re-seeded into “complete culture media” containing Nutristem™ XF Basal Media (Biological Industries, Israel) supplemented with supplement media for further processing. MNCs were counted using a hematocytometer, and cell viability was evaluated using trypan-blue dye staining (Sigma-Aldrich, Israel). MNCs were washed and re-suspended with Nutristem XF™ complete media and seeded on 175-cm^2^ culture flasks precoated with Attachment Solution XF. The culture supernatant containing the non-adherent mononuclear cells was removed, and the adherent cells were gently washed with 100 ml of DPBS. The medium was replaced twice a week, with fresh complete NutriStem™ XF growth medium until the culture reached 80–90% confluency but for no more than 12 days. Cells were subcultured at regular intervals, when the culture reached 80–90% confluence. Each subculture cycle was counted as a new passage. The cultures were cultured and subcultivated until reaching desired cell numbers (usually not more than three passages until cryopreservation).

A few days before cryopreservation, cells were characterized by FACS for human MSC markers and a biopotency test of mixed lymphocyte reaction (MLR). At the end of the process before cryopreservation, cells were tested for sterility, mycoplasma, and endotoxins. Cell were released for treatment upon receiving the results of the tests and according to the release criteria. Each cell batch was released with a certificate of analysis document (CoA).

Patients were initially treated (first treatment cycle) with 1 ×10^6^ MSCs per kg of body weight, intrathecally (via a standard lumbar puncture), and with the same number of MSCs intravenously. The scheduled treatment protocol was intended to include additional combined IT + IV injections every 6 months for up to 4 years. However, due to limitations in the number of cultured cells or the unwillingness of the patients to undergo repeated lumbar punctures (and additional bone marrow harvesting), the treatment was modified in most of the cases to single IV injections, or the time intervals between the injections were extended. An additional reason for this extension of the time intervals between the injections (up to 12 months) was related to the difficulties in traveling arrangements for many of the included patients who came from abroad. The duration of the study was 4 years and the median follow-up period was 27.8 months.

### Immunological Evaluation

Immunological analysis of peripheral blood mononuclear cells (PBMC) obtained from the treated patients was performed at baseline (before first treatment), after 4 h, at 1 day, and at 1, 3, and 6 months posttreatment, during the 6 month period following the first MSC transplantation. Specifically, the following tests were performed:

#### FACS Analysis of Lymphocyte Subsets

PBMCs were isolated by Histopaque-1077 (Sigma Aldrich, USA) density gradient centrifugation and, after gating for CD3 positivity, were stained with anti-CD4 PE, anti-CD25FITC (BD Biosciences, USA), anti-CD69 and anti-FoxP3 for FACS fluorescence cytometry. After gating for Lin-negativity, the isolated PBMCs were also stained for the myeloid dendritic markers CD11c and CD86PE (eBioscience, USA). The data were analyzed with a Beckman Coulter flow cytometer.

#### Lymphocyte Proliferation in Response to Phytohemaglutinin

The assay was carried out in 96-well, flat-bottomed Nunc plates (Daniel Biotech, USA). Lymphocytes were isolated from whole blood by Histopaque-1077 (Sigma Aldrich, USA) density gradient centrifugation and seeded at 2 ×10^5^/well in RPMI/10% FCS, 1 mM glutamine, and a penicillin–streptomycin mixture (Biological Industries) and stimulated with the lectin phytohemagglutinin (PHA) 1 mg/ml (Sigma Aldrich). Cultures were incubated for 48 h in a humidified atmosphere of 5% CO_2_ at 37°C, and then proliferation was assayed by 1 μCi/well of 3H thymidine (Amersham, UK) uptake. After 18 h of incubation with ^3^H thymidine, cells were frozen in −20°C and then harvested on fiberglass filters using a cell harvester (Skatron, Norway); radioactivity was measured by standard scintillation technique. The “Stimulation index” was calculated as the ratio between activated and non-activated cells.

## Results

### Safety

In general, there were no serious side effects during the whole 4 year duration of the study. Forty-one adverse events were registered (13 of them of moderate and 28 of mild severity). Thirteen of the patients experienced side effects of any kind. Eleven suffered from headache, six had transient low-grade fever, and three had backache. All these events resolved 1–7 days following the infusions. The full list of adverse events in each patient and each treatment is shown in Table 2. Interestingly, at those time points where patients were treated only intravenously with MSCs, there were no side effects at all (0). All the observed adverse events occurred in association with either intrathecal or combined IT + IV treatment.

**Table 2:** Safety (adverse events).

**Pts**	**No of tx**	**Route of administration**	**Intervals (months)**	**Adverse events**	**Severity**	**Outcome**
001	3	1—IV+IT 2—IV+IT 3—IV+IT	0 6 12	1—none 2—none 3—none		
002	3	1—IV+IT 2—IV+IT 3—IV+IT	0 12 24	1—headache, fever 2—headache 3—none	1—mild 2—moderate	1—resolved, 24 h 2—resolved, 3 days
003	4	1—IV+IT 2—IV+IT 3—IV 4—IV	0 12 24 36	1—fever, headache, general weakness 2—headache, general weakness 3—none 4—none	1—mild 2—mild	1—resolved, 3 days 2—resolved, 3 days
004	8	1—IV+IT 2—IV+IT 3—IV+IT 4—IV+IT 5—IV+IT 6—IV+IT 7—IV 8—IV+IT	0 6 12 18 24 30 36 42	1—headache, back pain 2—none 3—headache 4—back pain 5—none 6—none 7—none 8—headache	1—moderate 3—mild 4—mild 8—mild	1—resolved, 2 days 3—resolved, 24 h 4—resolved, 3 days 8—resolved 2 days
005	8	1—IV + IT 2—IV 3—IV 4—IV 5—IV 6—IV 7—IV 8—IV	0 6 12 18 24 30 36 42	1—none 2—none 3—none 4—none 5—none 6—none 7—none 8—none		
006	6	1—IV + IT 2—IV + IT 3—IV + IT 4—IV 5—IV + IT 6—IV + IT	0 6 12 18 24 36	1—neck rigidity, headache, back pain, leukocytosis 2—headache, back pain 3—none 4—none 5—headache 6—headache	1—severe 2—moderate 5—mild 6—mild	1—resolved, 3 days 2—resolved, 4 days 5—resolved 24 h 6—resolved, 24 h
007	6	1—IV + IT 2—IV 3—IV 4—IV 5—IV 6—IV + IT	0 12 18 24 36 42	1—fever 2—none 3—none 4—none 5—none 6—headache	1—moderate 6—mild	1—resolved 24 h 6—resolved 24 h
008	3	1—IV + IT 2—IV 3—IV	0 12 24	1—none 2—none 3—none		
009	4	1—IV + IT 2—IV + IT 3—IV + IT 4—IT	0 6 18 24	1—headache 2—fever, headache 3—none 4—none	1—mild 2—moderate	1—resolved 24 h 2—resolved 24 h
010	3	1—IV + IT 2—IV 3—IV	0 12 24	1—headache 2—none 3—none	1—mild	1—resolved 24 h
011	2	1—IV + IT 2—IV	0 12	1—none 2—none		
012	5	1—IV + IT 2—IV + IT 3—IV + IT 4—IV + IT 5—IV + IT	0 6 18 30 42	1—none 2—fever 3—none 4—headache 5—none	2—mild 4—mild	2—resolved 24 h 4—resolved 3 days
013	5	1—IV + IT 2—IV + IT 3—IV + IT 4—IV + IT 5—IT	0 6 12 24 36	1—urinary retention, fever, headache 2—none 3—none 4—none 5—none	1—moderate	1—resolved, 24 h
014	2	1—IV + IT 2—IV	0 12	1—none 2—none		
015	3	1—IV + IT 2—IV + IT 3—IV + IT	0 12 24	1—none 2—headache 3—none	2—mild	2—resolved 24 h
016	3	1—IV + IT 2—IV + IT 3—IV	0 12 24	1—none 2—none 3—none		
017	2	1—IV + IT 2—IV + IT	0 6	1—fever 2—headache	1—mild 2—mild	1—resolved 24 h 2—resolved 24 h
018	2	1—IV + IT 2—IV	0 6	1—none 2—none		
019	2	1—IV + IT 2—IT	0 6	1—none 2—none		
020	6	1—IV + IT 2—IV + IT 3—IV 4—IV + IT 5—IV + IT 6—IV + IT	0 6 18 24 36 42	1—none 2—back pain 3—none 4—headache, fever 5—none 6—headache	2—mild 4—mild 6—mild	2—resolved, 3 days 4—resolved 24 h 6—resolved 24 h
021	3	1—IV + IT 2—IV + IT 3—IV + IT	0 12 24	1—none 2—none 3—none		
022	2	1—IV + IT 2—IV + IT	0 6	1—none 2—none		
023	2	1—IV + IT 2—IV + IT	0 12	1—back pain 2—back pain, sciatic pain	1—mild 2—moderate	1—resolved, 3 days 2—resolved 7 days
024	2	1—IV + IT 2—IV	0 6	1—none 2—none		
	**Total: 89**	**IV = 86 injections** **IT = 64 injections** **IT + IV: 61**		**41 adverse events**	**1 severe,** **13 of moderate severity, others: mild**	**All resolved** **between 1 and 7 days**

*Tx = treatments with MSC; Pts = patients*.

The definition of the severity of adverse events was according to FDA recommendations; a severe adverse event was any event leading to hospitalization. The single severe event in our study was a case with neck rigidity and back pain, who was hospitalized with suspected meningitis, which was ruled out. The patient was discharged 2 days later.

### Clinical Effects

**Figure 2:**
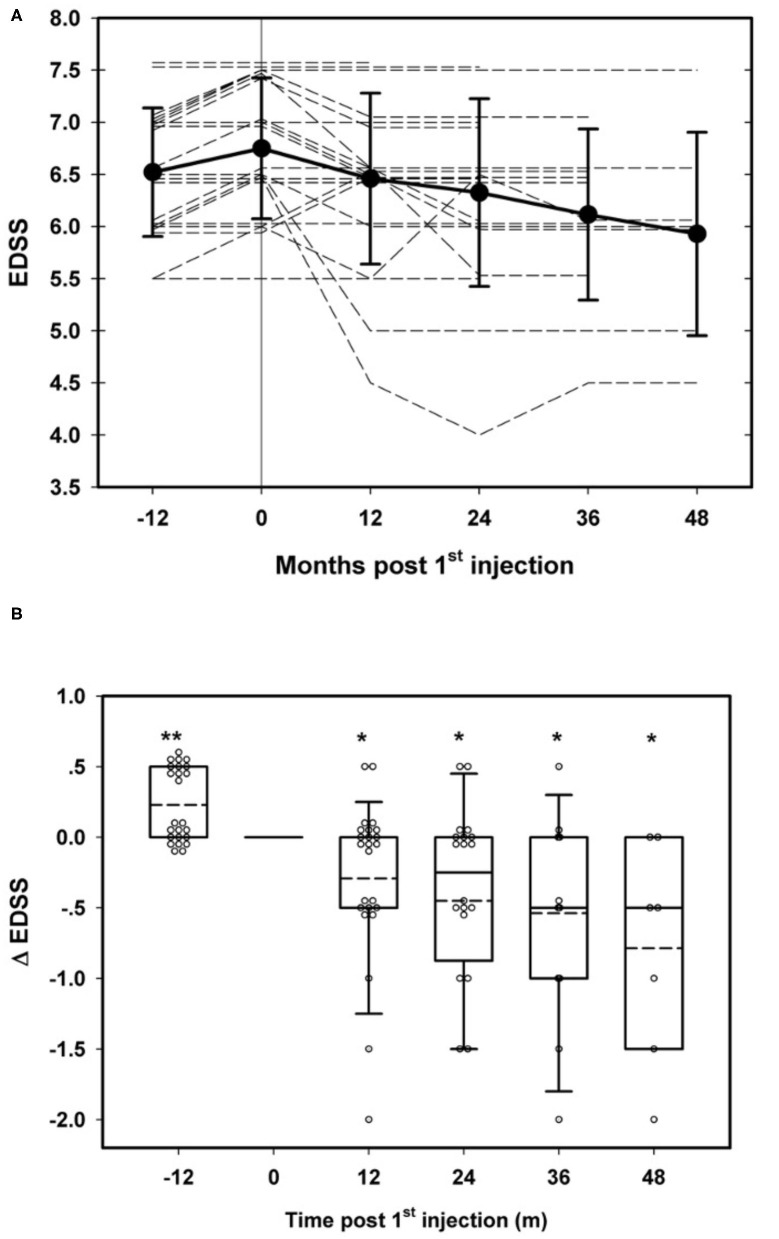
Long-term clinical effects of repeated transplantation of mesenchymal stem cells (MSCs) in multiple sclerosis (MS). **(A)** Changes in EDSS in individual patients before and after MSC transplantation. **(B)** Rate of EDSS change before and after MSC transplantations. *p < 0.05, **p < 0.01, at the respected time points vs baseline values (Wilcoxon signed rank test).

**Figure 3:**
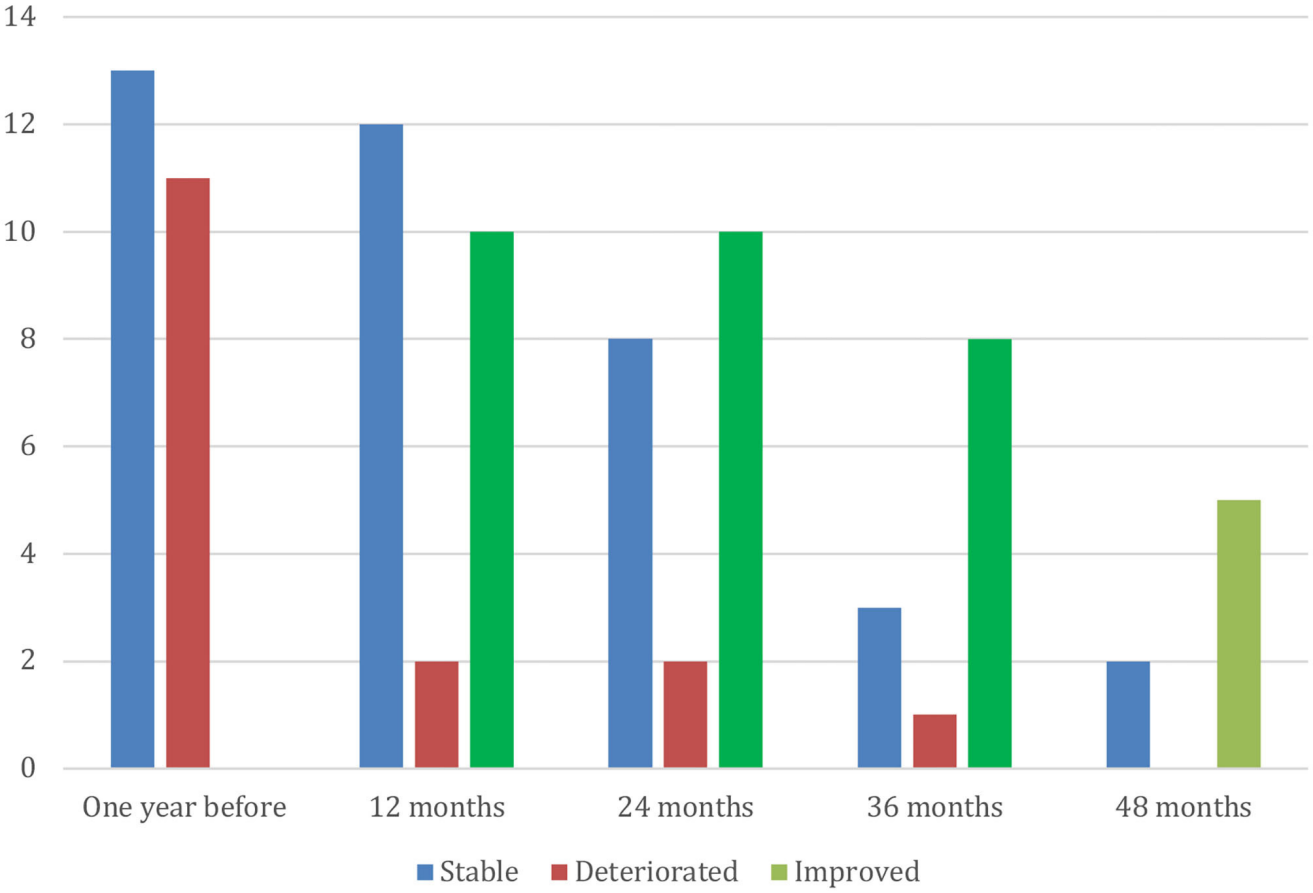
Number of patients with improvement or deterioration in EDSS compared with baseline.

Twenty-two of the 24 patients were either stable or improved in the EDSS score at the last follow-up visit. Ten patients had a lower than baseline EDSS score at last follow-up (nine were among those who received more than two treatments and one in the subgroup of two treatments or less, *p* = 0.04, chi-square test) (Table 3). The mean EDSS score reduced from 6.75 ± 0.68 at baseline to 6.42 ± 0.84 at the last visit (*p* = 0.028, Wilcoxon ranked sign test), during a mean follow-up period of 29.24 ± 12.76 months (range: 12–59.5) (Figure 2 and Table 3). The mean change in EDSS in the year prior to inclusion was +0.27 ± 0.25 and −0.35 ± 0.63 (*p* = 0.002, Wilcoxon sign ranked test) at the end of follow-up (last visit) (Figure 2 and Table 3). The numbers of patients who were stable, improved, or deteriorated in EDSS, each year, are shown in Figure 3.

**Table 3:** Long-term clinical effect of multiple MSC transplantations.

**Patient**	**No. of Tx**	**1 year before**	**EDSS Baseline**	**12 months**	**24 months**	**36 months**	**48 months**
001	3	6.5	6.5	6.5	6.5		
002	3	5.5	6.0	6.5	6.5	6.5	
003	4	6.0	6.0	6.0	6.0	6.0	
004	8	6.0	6.5	4.5	4.0	4.5	4.5
005	8	6.5	6.5	5.0	5.0	5.0	5.0
006	6	6.5	7.0	6.5	6.5	6.5	6.5
007	6	7.0	7.0	6.5	6.0	6.0	6.0
008	3	7.0	7.5	7.5	7.5	7.5	7.5
009	4	7.5	7.5	7.5	7.5		
010	3	7.0	7.0	6.5	6.5	6.5	
011	2	7.0	7.0	7.0	7.0		
012	5	6.0	6.0	5.5	6.5, R^*^	6.0	6.0
013	5	7.0	7.5	7.0	7.0	7.0	
014	2	5.5	5.5	5.5	5.5, R^*^		
015	3	7.0	7.5	7.5	7.5		
016	3	7.0	7.5	6.5	6.0		
017	2	7.0	7.5	7.5			
018	3	6.0	6.0	6.5, R^*^			
019	2	5.5	5.5	5.5			
020	6	6.0	6.5	6.0	6.0	6.0	6.0
021	3	6.0	6.5	6.5	5.5	5.5	
022	2	7.5	7.5	7.5			
023	2	6.5	6.5	6.5	6.5		
024	2	7.0	7.5	7.0	7.0		
**Mean ± SD**		**6.52 ± 0.62**	**6.75 ± 0.68**	**6.46 ± 0.82**	**6.33 ± 0.88**	**6.08 ± 0.82**	**5.93 ± 0.98**

*At last follow-up (for all 24 patients): 6.42 ± 0.84.*

* *R, relapse during the study*.

Although the aim of our study—in terms of clinical effects—was to follow-up changes in disability in patients with progressive disease, we noticed that 14 of the patients had activity expressed by superimposed relapses during the year prior to inclusion to the study (total numbers of relapses 16). During the period of MSC treatments, only three relapses were noted in three patients (*p* = 0.002, Wilcoxon signed rank test, compared with the year prior to treatment) (Table 3).

### Immunological Effects

#### Effect of Mesenchymal Stem Cell Treatment on the Proportions of Various Immune Subpopulations

**Figure 4:**
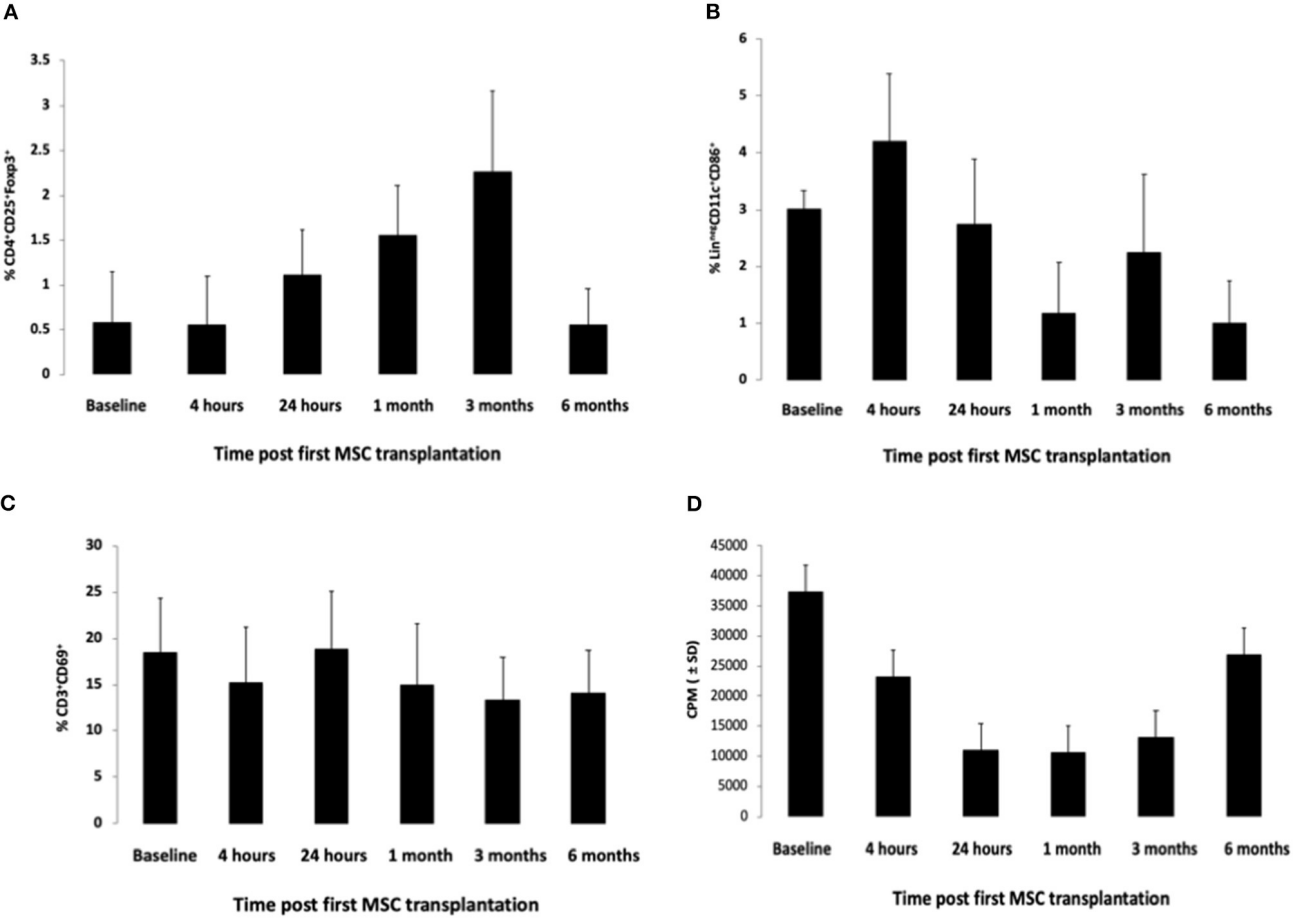
Immunological changes following MSC transplantation at various time points during the 6 months following the first MSC transplantation. **(A)** Changes in CD3-gated, CD4+CD25^high^+Foxp3+ cells (mostly T-regs), at various time points following the first MSC transplantation. *P* = 0.002 at 4 h vs. baseline, p = 0.0034 at 24 h, *p* = 0.002 at 1 month, *p* = 0.0007 at 3 months, nonsignificant at 6 months (Wilcoxon signed rank test) (*n* = 8). **(B)** Changes in Lin-CD11c+CD86+ cells (representing antigen-presenting cell populations, mostly dendritic cells, but also macrophages), at various time points following the first MSC transplantation. *P* = 0.0002 at 1 month vs. baseline, others not statistically significant (Wilcoxon signed rank test) (*n* = 8). **(C)** Changes in CD3+CD69+ cells (mostly activated lymphocytes), at various time points following the first MSC transplantation. *P* = 0.029 at 3 months vs. baseline, others not statistically significant (Wilcoxon signed rank test) (*n* = 8). **(D)** Changes in the *ex vivo* proliferative ability of lymphocytes upon stimulation with phytohemagglutinin (PHA), at various time points following the first MSC transplantation. *P* = 0.001 at 4 h vs. baseline, *p* = 0.0002 at 24 h, *p* = 0.0009 at 1 month, *p* = 0.003 at 3 months, not statistically significant at 6 months (Wilcoxon signed rank test) (*n* = 8).

Immunological follow-up showed a statistically significant upregulation of the CD4+CD25^high^+FoxP3+ cells (3-fold at month 1 and 4-fold at 3 months), a population representing the majority of T-regulatory cells (T-regs). At 6 months, these proportions returned to baseline values (*p* = 0.002 at 4 h vs. baseline, *p* = 0.0034 at 24 h, *p* = 0.002 at 1 month, *p* = 0.0007 at 3 months, non-significant at 6 months, Wilcoxon signed rank test) (*n* = 8) (Figure 4A).

Other changes included a transient reduction in the proportion of Lin^−^CD11c^+^CD86^+^ cells, representing antigen-presenting cell populations (mostly dendritic cells but also macrophages) (from 2.97 to 1.2% at 1 month), following MSC transplantation, indicating a possible downregulatory effect on the antigen presentation process and a mild reduction in the proportion of CD3+CD69+ cells, which was more significant at month 3 (Figures 4B,C).

#### Effect of Mesenchymal Stem Cell Treatment on the Proliferation Ability of Lymphocytes

Following *ex vivo* stimulation of peripheral blood lymphocytes (obtained from the patients at various time points following the first MSC transplantation) with the phytohemaglutinin (PHA), there was a 71% decrease in the proliferative cell response at 24 h, 72% decrease at 1 month, 65% at 3 months, and 28% at 6 months (*p* = 0.001 at 4 h vs. baseline, *p* = 0.0002 at 24 h, *p* = 0.0009 at 1 month, *p* = 0.003 at 3 months, not statistically significant at 6 months) (Wilcoxon signed rank test) (*n* = 8) (Figure 4).

## Discussion

In this open trial, repeated intrathecal and intravenous administration of MSCs in 24 patients with progressive MS, not responding to the conventional immunomodulatory treatments was shown safe at the short/intermediate term. During the observation period of up to 4 years, there were indications of clinical benefits (i.e., stabilization or improvement in EDSS score), especially in patients treated with more than two injections. Although this was predominantly long-term safety study significant clinical benefits of the MSC treatments, were detected. At the end of the follow-up period, 22 out of the 24 patients treated with MSC had a stabilized had a stabilized or improved EDSS and were defined as “long-term responders”. During the 6 months following the first treatment course, immunomodulatory effects of the treatment were also detected, as indicated by an increase in the proportion of the CD4+CD25+FoxP3+ cells (mostly representing the T-regs population) (peaking at 1 day and lasting up to 1–3 months post-transplantation), a transient downregulation of the proliferation ability of the lymphocytes (lasting for up to 3 months) and a moderate downregulation of the CD3+CD69+ and Lin-CD11c+CD86+ cells, representing mainly the activated lymphocytes and antigen-presenting cell populations (mostly dendritic cells and macrophages). Immunological analysis was performed only during the first cycle of treatment, since the subsequent treatments were not given at the same time points in each patient, and therefore, cumulative immunological effects of the repeated treatments could greatly vary among the patients and could complicate the interpretation of the findings.

Despite the development of highly efficient and more targeted immunotherapies for MS, two major unmet needs still exist: (1) the need for treatment to suppress compartmentalized and meningeal inflammation in the central nervous system (CNS), which seems to drive tissue injury and progression of disability (22–24). These compartmentalized inflammatory and degenerative activities seem to be less responsive to the majority of immunomodulatory drugs, accounting for the relatively poor efficacy of the majority of registered MS therapies in progressive MS, with minor exceptions (25, 26).

(2) The need for a treatment that may substantially promote regeneration–remyelination. Generally, the CNS loses its capacity for efficient regeneration and remyelination over time. This is especially pronounced in chronic neuroinflammatory and neurodegenerative diseases such as MS, possibly due to an insufficiency of growth factors or defective mobilization of intrinsic CNS stem cells/oligodendrocyte progenitors (27–29).

Based on their described properties (4, 30–32), stem cells may represent a “logical” treatment approach to achieve those unmet needs and possibly induce neuroprotection and enhance endogenous remyelination (as indicated by animal studies). Moreover, stem cells are strong immunomodulators (6, 29, 33–35) that may potentially downregulate the localized and compartmentalized inflammation upon their migration to the CNS (22, 24). Several studies have shown that embryonic, neuronal, and other adult stem cells can induce beneficial clinicopathological effects in animal models of neurological diseases, including MS (3, 7–9, 36–39). MSCs are the most commonly used type of stem cells for such cell-based therapies, as they have the following practical advantages for clinical use over other types of stem cells: (1) They can be easily cultured and expanded in large quantities. (2) They can be obtained from the patient, thus, eliminating the need for a donor, the risk of rejection, or the need for chemotherapy. (3) They seem to be safe and carry low risks of malignant transformation. During the last decade, MSC treatments have been applied to various neurological diseases in small or pilot open-label trials (10–18, 40), with promising indications.

The putative mechanism of action of MSC in neurological diseases is controversial. Some investigators claim that the most prominent effects are mediated through peripheral immunomodulation (6, 29, 34, 35). Our group has long advocated that neuroprotective and neurotrophic mechanisms play the most crucial role, as supported by our findings in animal models and pilot trials (4, 13, 17) and that the intrathecal way of administration, which brings a higher proportion of the injected cells into close proximity with damaged areas of the CNS, is preferable to the intravenous injection. Indeed, the findings of our recent double-blind randomized trial in MS (19) showed that the intrathecal injection of MSC was superior to the intravenous at several parameters.

Concerning the (rather short lasting) immunological changes that were shown in the current study, they seem—most probably—to be caused by the intravenous administration of the MSCs, since most of the intravenously administered MSCs have been shown to reside in the periphery and not the CNS (41). Although it is difficult to estimate the clinical relevance of the observed immunological changes, they may have a possible impact on the autoimmune responses of lymphocytes that target myelin antigens and, therefore, be beneficial for MS. Moreover, if the MSCs indeed (via the intrathecal route) migrate to the areas of CNS lesions, they could theoretically downregulate locally the compartmentalized inflammation, potentially acting as “Trojan horses.”

On the other hand, downregulation of either antigen-presenting cells or the activation cascade of immune cells and upregulation of regulatory cells may introduce potential risks, such as increased risk for carcinogenesis. Although such risks theoretically exist, they do not seem to be substantial, since these immunomodulatory effects that were induced by the MSCs were transient and rather short lasting, in our study.

In any case, peripheral immunomodulation alone does not seem to sufficiently explain the wide range of clinical beneficial effects induced by MSC transplantation, which were observed in our previous and the current trial (13, 17, 19, 31).

The strengths of our trial include the inclusion of patients with progressive MS, in which conventional immunotherapies were shown ineffective, the long follow-up (up to 4 years), the treatment protocol of repeated (up to eight) administrations of stem cells, and the robust clinical benefits observed in disability progression. The main limitation of our study is obviously related to the small number of patients and the open-label design. Additional limitations of this trial are related to the inclusion of a non-homogenous patients' population (with different types of progressive MS and disease duration) and the lack of uniformity in the treatment protocol (number of injections and intervals between them), for the reasons that are explained in the *Methods* section.

Another possible problem in the interpretation of our findings could be related to the fact that half of our patients had a deterioration in the EDSS score during the year prior to inclusion. Part of the beneficial effects, therefore, could be theoretically related to a “regression to the mean” phenomenon. However, such regression, although may have affected the clinical changes at some degree (especially in the first months of the study), cannot—to our view—explain the findings of the benefits during the subsequent cycles of treatment and the long-lasting clinical improvements.

In conclusion, in our present, open trial, we showed that repeated intrathecal administrations of MSCs in patients with progressive MS was safe at the short/intermediate term and induced clinical benefits (especially in patients treated with more than two injections) that lasted for up to 4 years and included stabilization of the progression of MS and improvements of neurological disability, paralleled by short-term immunomodulatory effects. The data presented here may help in the design of larger trials that could further evaluate the clinical potential of repeated injections of MSCs in MS and other neurological and neuroimmunological diseases.

## Data Availability Statement

The original contributions presented in the study are included in the article/supplementary material, further inquiries can be directed to the corresponding author/s.

## Ethics Statement

The studies involving human participants were reviewed and approved by Hadassah Ethics committee. The patients/participants provided their written informed consent to participate in this study.

## Author Contributions

DK, PP, and IK participated in the writing of the manuscript. PP was the clinical PI of the trial. All authors participated in the organization and performance of the trial.

## Conflict of Interest

The authors declare that the research was conducted in the absence of any commercial or financial relationships that could be construed as a potential conflict of interest.
